# Patterns of Time Use across the Chronic Obstructive Pulmonary Disease Severity Spectrum

**DOI:** 10.3390/ijerph15030533

**Published:** 2018-03-16

**Authors:** Toby Hunt, Marie T. Williams, Timothy S. Olds, Dorothea Dumuid

**Affiliations:** Alliance for Research in Exercise, Nutrition and Activity (ARENA), Sansom Institute for Health Research, School of Health Sciences, University of South Australia, City East Campus, North Terrace, Adelaide 5000, Australia; marie.williams@unisa.edu.au (M.T.W.); timothy.olds@unisa.edu.au (T.S.O.); dorothea.dumuid@mymail.unisa.edu.au (D.D.)

**Keywords:** COPD, time use, BODE index

## Abstract

Descriptions of time use patterns in people with chronic obstructive pulmonary disease (COPD) are scarce and the relationship between use-of-time and COPD severity remains unclear. This study aimed to describe a typical day for people with COPD and to explore the differences in time-use patterns across the Body Mass-Index, Airflow Obstruction, Dyspnoea and Exercise Capacity (BODE) index using compositional analyses. Using a cross-sectional design, 141 adults with clinically stable COPD had their demographics, objective measures of function (pulmonary, exercise capacity and physical activity), and self-reported COPD-related impairment recorded. Daily time-use compositions were derived from 24-h accelerometry and 24-h use-of-time recall interviews. Compositional multiple linear regression models were used to explore the relationship between the BODE index and 24-h time-use compositions. These models were used to predict daily time (min/d) that is spent in time-use components across the BODE index. The BODE index score was clearly associated with 24-h accelerometry (*p* < 0.0001) and 24-h use-of-time recall (*p* < 0.0001) compositions. Relative to the remaining time-use components, higher BODE index scores were associated with greater sedentary behaviour (*p* < 0.0001), Quiet time (*p* < 0.0001), Screen time (*p* = 0.001) and Self-care (*p* = 0.022), and less daily Chores (*p* < 0.0001) and Household administration (*p* = 0.015) time. As the BODE index scores increased, time-use predictions were strongly associated with decreases in Chores (up to 206 min/d), and increases in Screen (up to 156 min/d) and Quiet time (up to 131 min/d). Time–use patterns may provide a basis for planning interventions relative to the severity of COPD.

## 1. Introduction

The BODE (Body Mass-Index, Airflow Obstruction, Dyspnoea and Exercise Capacity) index is a multi-component grading system primarily developed as a predictor of respiratory and all-cause mortality risk in people with chronic obstructive pulmonary disease (COPD) [1,2]. Since its development, it has become used as a clinical outcomes marker for disease severity [3,4], pulmonary rehabilitation responsiveness [5,6], and hospitalizations due to exacerbations [7].

COPD is a progressive respiratory disease, which is characterised by increasing functional limitation and exertional dyspnea [8]. Functional limitations appear early in the course of the disease [9,10,11]. People with COPD exhibit significant reductions in both the duration and intensity of physical activities when compared to healthy controls [12,13,14], and little if any moderate to vigorous physical activity (MVPA) on a typical day [9,15,16]. To date, activity research in people with COPD has largely focused on quantifying the time spent in differing intensities of physical activity. Few studies have explored the type and context of activities or time spent in other activity domains and no published studies have explored the 24-h time use of people with COPD [17]. The absence of time-use data in this clinical population means that comprehensive activity profiles are also absent, limiting our understanding of how people with COPD spend their time and how disease severity impacts activity patterns.

Activity profiles are bounded in time (there are only 24 h per day), meaning that activity domains are co-dependent (correlated). If the time in one domain is decreased, this must be compensated by increases in other domains. This constitutes a special class of data that should be analysed using techniques that recognise these constraints. Analysis of compositional data (compositional data analysis, or CoDA), acknowledges the bounded nature of activity profiles and takes into account the relative nature of time-use data. This study aimed to (1) describe a typical day for people with COPD using both energy expenditure bands and use-of-time recall data; and, (2) explore relationships between COPD severity (BODE index) and time use using CoDA. We hypothesised that as severity of COPD increased, individuals would spend less time in physically-demanding activities, and more time in sedentary tasks.

## 2. Materials and Methods

### 2.1. Methods

This study presents a cross-sectional analysis of baseline data from a randomised controlled trial (RCT) when comparing participation in a comprehensive pulmonary rehabilitation program (CPRP) with and without a cognitive behavioural therapy program for breathlessness [18] and a cohort of usual care participants. Participants enrolled in the RCT were referred to Repatriation General Hospital’s (RGH) CPRP between 2011 and 2014, while the usual care participants were recruited using convenience sampling from RGH’s existing clinical and research databases throughout 2014 and 2015. Ethical approvals were obtained from the University of South Australia Human Research Ethics Committee (protocol number: 153/07) and the Southern Adelaide Clinical Human Research Ethics Committee (protocol number: 56/07). Written informed consent was obtained from all participants before any study procedures were undertaken.

People with clinically stable COPD and airflow limitation of at least grade 2, according to the Global Initiative for Chronic Obstructive Lung Disease (GOLD) (i.e., post bronchodilator forced expiratory volume in 1 s (FEV_1_) < 80% predicted, forced expiratory ratio (forced expiratory volume in 1 s/forced expiratory volume, FEV_1_/FVC) < 0.7) [19] were included in this study (*n* = 141, age 70.6 y, Females *n* = 57, FEV_1_% pred 49.9%). Participants were excluded if they had: (a) significant cognitive impairment (Mini-Mental State Examination score < 23); (b) clinically unstable COPD (e.g., worsening of respiratory symptoms within the preceding four weeks); (c) co-morbidities likely to render exercise unsafe; or, (d) were currently registered for lung volume reduction surgery or lung transplantation. In addition, usual care participants were excluded if they had been enrolled in any form of a CPRP in the preceding two years.

### 2.2. Research Procedure

Identical research protocols were used for all of the participants. Each participant attended one in-clinic session during which demographics (age, sex, body mass index [BMI], and residential postcode), objective measures of function (pulmonary and exercise capacity), and self-reported measures of COPD-related impairment (comorbid health conditions, exertional breathlessness) were collected. Participants then completed a seven-day activity-monitoring protocol during which patterns of habitual activity were recorded using an accelerometer and two, 48 h use-of-time recall interviews.

### 2.3. Outcome Variables and Measurement Protocols

All the variables of interest were derived either directly or as composite scores from use-of-time data (e.g., use-of-time recall or accelerometry data), COPD assessments (e.g., spirometry, 6 min walk test [6MWT], modified Medical Research Council dyspnoea scale [mMRC]) and/or retrospective health status data reviews (e.g., COPD-specific co-morbidity test [COTE] index score [20]). Residential postcodes obtained during the clinic visit were used as a measure of socio-economic status (Index of Relative Socio-economic Disadvantage [IRSD]) [21].

MARCA use of time recalls—The Multimedia Activity Recall for Children and Adults (MARCA) is a use-of-time tool that has been validated in older adult and COPD populations [15,22]. Using structured telephone-based interviews, participants systematically recall individual activities undertaken during the two preceding days. Activities in the MARCA’s compendium are linked to energy expenditure estimates [23] and using hierarchical structures are collapsible into “superdomains” of like activities (e.g., Chores, Screen time or Self-care) or energy expenditure bands (e.g., sleep, sedentary, light physical activity [LPA] and moderate to vigorous physical activity [MVPA]), which together represent the entire day. 

In people with COPD the MARCA has been shown to have high test-retest reliability for all major health-related time use categories (ICCs > 0.88) and modest to strong agreement with MVPA and accelerometry-derived physical activity level estimates (r = 0.43–0.80) [12].

Accelerometry—Accelerometry data were collected using a seven-day protocol. Participants wore an Actigraph GT3X+ accelerometer on their dominant hip for seven consecutive days, removing devices only for water-based activities. Non-wear and sleep were logged in paper diaries. Accelerometers recorded activity in 10-s epochs at a frequency of 70 Hz. Minimum wear compliance was four days (three weekdays and one weekend day), each consisting of at least 10 h of valid data. Troiano et al.’s [24] activity cut-points as well as the widely accepted 100 counts/min for sedentary behaviour [25], were used to define the energy expenditure bands. Actigraph GT3X+ accelerometers show modest agreement with energy expenditure estimates obtained from doubly labelled water (r = 0.30) [26], and strong agreement with indirect calorimetry in people with COPD (r ≥ 0.77) [27]. For both MARCA and accelerometry data weekday-weekend weightings (5:2 ratio) were applied.

Physiological impairment data—Pulmonary function testing was conducted using a Jaeger MS-PFT (Jaeger, Germany). The maximum six-minute walk distance (6MWD) obtained from duplicate 6MWTs was recorded. All of the testing was conducted in accordance with recommended international standards [1,28,29]. BODE index scores were calculated from the relevant individual components, [1] and a medical chart review was conducted to calculate each participant’s COTE index score [20].

### 2.4. Statistical Analysis

Time (min/d) spent in energy expenditure bands and MARCA superdomains were first analysed descriptively using traditional statistics (arithmetic means and standard deviations). Compositional data analysis (CoDA) was undertaken in R (R Development Core Team, Vienna Austria), using the statistical packages *Compositions* [30] and *zCompositions* [31]. Three compositions were created for each participant. Two used energy expenditure data (min/d in sleep, sedentary time, LPA and MVPA) collected from accelerometry (Composition 1) and MARCA recalls (Composition 2). The third composition was constructed using MARCA use-of-time data (min/d in Chores, Household administration, Quiet time, Screen time, Self-care, Sleep, Socio-cultural, and Sports/exercise) (Composition 3).

According to the principles of CoDA, the compositions were expressed as a set of isometric log-ratio (*ilr*) coordinates. As the logarithm of zero is undefined, the presence of zero values in any component precludes the application of *ilr*. Where participants recorded no time in one or more components zero values were classed as below detection limits, and replaced using small non-zero values (e.g., 65% of the epoch length) as recommended in Martin-Fernandez [32].

Compositional descriptive statistics were produced for the center and dispersion of each composition. Compositional means were calculated as the geometric means of each profile’s compositional parts (e.g., energy expenditure bands for energy expenditure compositions) and were linearly adjusted, so that all the parts together summed to the whole day (1440 min) [33].

Multiple linear regression models were created for each composition with BODE index score (explanatory variable) and *ilr* co-ordinates (dependent variables). Models were adjusted for COTE index score, IRSD, smoking status, age, and sex. First, an omnibus type III MANOVA test of the model fit was used to test whether the BODE index was associated with the complete set of *ilr* coordinates (i.e., the daily time-use composition). Where model fit was acceptable, a specific type of *ilr* transformation, as described in Chastin et al. [34] was used to construct the dependent *ilr* coordinate. This *ilr* transformation produces a “*pivot coordinate*”, which has one component in its numerator, and the geometric mean of the remaining components in its denominator. Thus, the pivot coordinate represents one time-use component, relative to the remaining components. The regression parameters for a given BODE index score, returned for this pivot coordinate, represent the relationship between the given BODE index score and one component, relative to all of the remaining components (e.g., if the pivot coordinate contains sleep, relative to the remaining components, a positive beta suggests that as the BODE index score increases, sleep is predicted to increase, while the remaining time-use components all decrease in equal proportions to maintain the daily total of 1440 min). Pivot coordinates were constructed for each time-use component. The pivot coordinates were iteratively used in the linear model to assess the relationship between the BODE index score and each component, relative to the remaining components.

Finally, to quantify the association between the BODE index scores and the compositions, the complete multiple linear regression models (with the full set of ilr coordinates) were used to predict new sets of *ilr* coordinates for each score across the BODE index (i.e., 0 through 10) [35]. The *ilr* coordinates were back-transformed to compositions (min/d). Results were graphed to aid interpretation.

### 2.5. Power Analysis

This design was capable of detecting a small effect size (Cohen’s f^2^) of 0.06 based on post hoc calculations using this study’s sample size (*n* = 135), five covariate predictors, a power of 80%, and an alpha of 0.05.

## 3. Results

**Figure 1:**
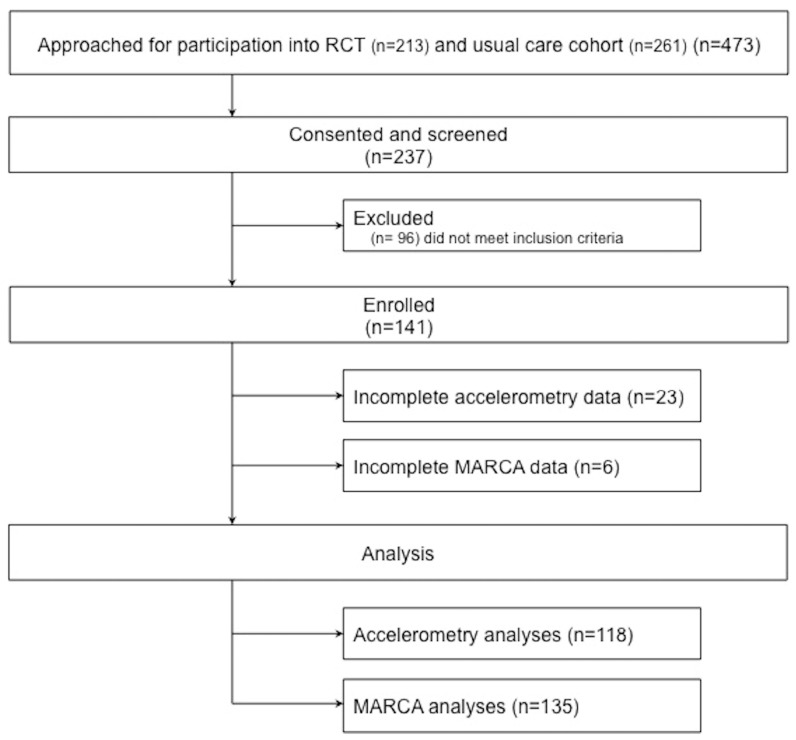
Participant flow—cross-sectional use of time study. RCT—randomised controlled trial, n—number, MARCA—Multimedia Activity Recall for Children and Adults.

The participant flow of this cross-sectional study is presented in Figure 1. Baseline descriptive data for socio-demographic, physiological impairment, COPD-related exertional breathlessness, and disease status are provided in Table 1.

### 3.1. Descriptive Exploration of Habitual Activity Patterns

Energy expenditure bands—Both accelerometry and MARCA data indicated people with COPD spent the majority of their days engaged in sedentary behaviours (51% and 47%, respectively). Sleep accounted for approximately a third of the day (31% and 33%, respectively) and the remaining proportion of the day was spent almost exclusively in LPA (17% and 14%, respectively) with little engagement in MVPA (1% and 6%, respectively). No significant differences were observed between males and females.

**Figure 2:**
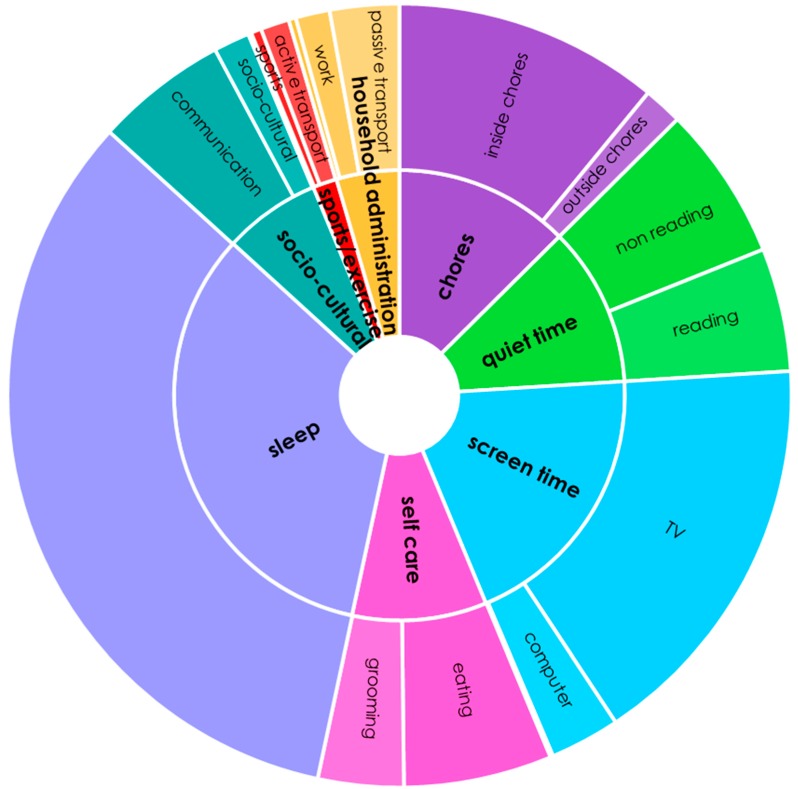
Composite activity profile presenting a typical profile of 24-h time use. Mean time spent in MARCA superdomains (in min/d) is presented using arithmetic means from baseline MARCA data.

MARCA use-of-time domains - Data obtained from the MARCA use-of-time recalls (Table 2 and Figure 2), indicate that people with COPD spent significant quantities of the waking day using screens (284 min/d) or sitting quietly (165 min/d). Three hours per day were spent doing chores, the majority of which were comprised of indoor chores. Minimal time was spent in formal sports or exercise (25 min/d), which was mostly comprised of walking (17 min/d). There were no significant differences between males and females.

### 3.2. Associations between Clinical Characteristics and Use of Time in People with COPD Using Compositional Analysis

The BODE index scores were significantly related to the time-use compositions (i.e., the *ilr* coordinate sets, omnibus *p* < 0.0001) in all three models. In Model 3, socio-economic status was also significantly associated with the compositional model (*p* = 0.025). The regression parameters returned for the pivot coordinate (representing the relative dominance of a particular component to the remaining components) are presented in Table 3. In both energy expenditure models, the BODE index was positively associated with sleep and sedentary time, and is negatively associated with MVPA, all being relative to the remaining parts (all *p* < 0.0001; Models 1 and 2). The BODE index was negatively associated with time spent in Chores and Sports/exercise, and positively associated with time spent in Quiet and Screen time (all *p* ≤ 0.0001; Model 3), all being relative to the remaining time-use domains.

**Figure 3:**
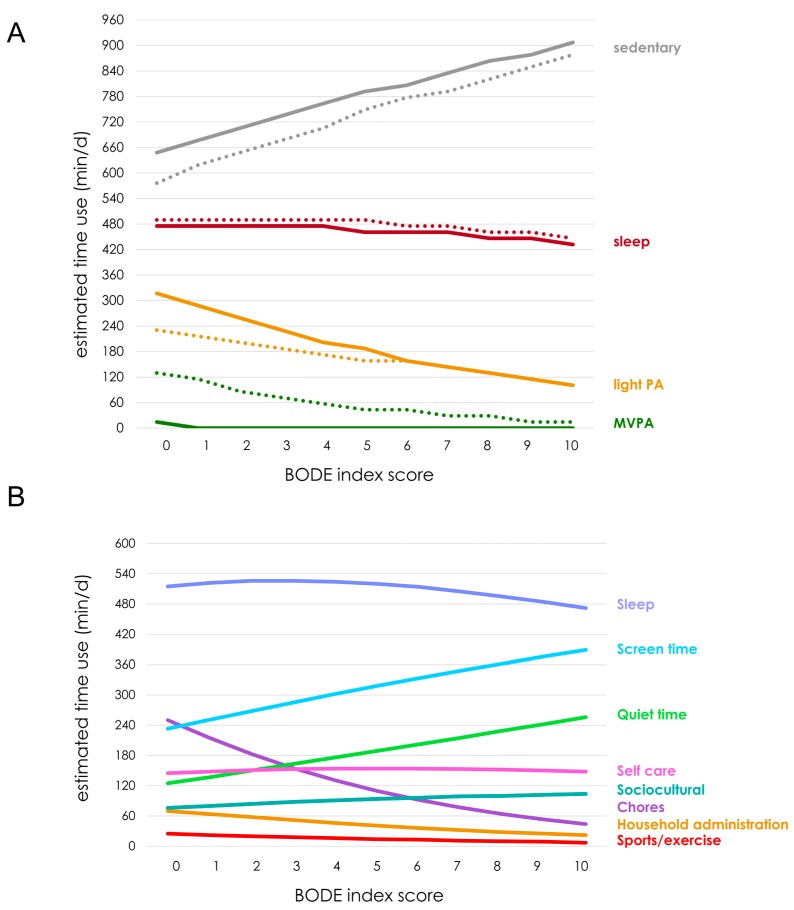
Estimated daily time use (in min/d) for components across the BODE index. BODE index—the Body mass, airflow Obstruction, Dyspnoea and Exercise index, Light PA—Light physical activity, MVPA—moderate to vigorous physical activity, min/d—minutes per day. Data are expressed as means derived from compositional models (i.e., geometric means of components, adjusted to collectively sum to a total of 1440 min/day). (**A**) Predicted time use using energy expenditure bands (accelerometry estimates are shown with solid lines, and MARCA estimates are shown with dashed lines); (**B**) Predicted time use using MARCA super domain categories.

Cross-sectional predictions of daily time-use composition across the BODE index (BODE 0 to BODE 10) are shown in Figure 3. Sedentary time was predicted to increase with increasing BODE index scores, totaling an additional 252 min/d at BODE 10 when compared to BODE 0. This increase was at the expense of light physical activity and sleep (Models 1 & 2). Total MVPA was predicted to be low across all the BODE index scores. Predictions for the use-of-time composition (Model 3) showed less time in Chores (totaling 206 min/d) and Household administration (totaling 48 min/d) across the BODE index, with compensatory increases in Quiet (131 min/d), Screen (156 min/d) and Sociocultural (28 min/d) time. Little time was devoted to physically demanding activities (max. 25 min/d), regardless of the predictive BODE index score. Predicted time in Self-care was similar across all of the BODE index scores.

## 4. Discussion

This study presents the first comprehensive analysis of use-of-time data in people with COPD using CoDA. The key findings of this study were: (1) people with COPD spent the majority of their waking day undertaking activities requiring limited energy expenditure; (2) Screen time, Chores, and Quiet time activities comprised most of the waking day; (3) strong and significant relationships existed between BODE index scores and time-use composition; and, (4) the most striking use-of-time pattern identified was the relative reduction in time spent doing Chores that are associated with increasing BODE index scores.

This study presents a clear and concise composite activity profile representing an average day for a South Australian with moderate to severe COPD (Figure 2). As consistent with previous literature, we found that people with COPD have large amounts of sedentary time and little or no engagement in MVPA [10,36,37] Despite a relatively narrow geographic recruitment region, our participants’ characteristics (age 70.6 years, FEV_1_% pred 49.9%) and the patterns of time use we identified were broadly comparable with those identified in a recent systematic review of use-of-time studies in people with COPD [17], where data was available from various global locations (e.g., North America, South America, and Europe) (median ages 65.3 y, median FEV_1_ %pred 45%). 

It is clear from our data and that of others that people with COPD exhibit patterns of activity distinct from general older adult populations [9,10,11,17,38] When compared with similar aged peers without COPD (mean 62.3 years), from a similar geographic location [38], people with COPD, regardless of BODE index score, which spent substantially less time in Sports/exercise and Sociocultural activities than their peers. Despite having similar quantities of Quiet and Screen-time at BODE 0, the presence of even low level impairment (as indicated by BODE index scores 1 or 2), was associated with substantial differences in predicted time-use patterns, with Quiet and/or Screen-time almost double, and time spent on Chores considerably less.

Our data also clearly demonstrate that distinct patterns of time use are associated with differing degrees of disease severity. Significant negative correlations were observed between the BODE index and LPA (e.g., Chores and Household administration) and/or sedentary activities (e.g., Screen time and Quiet time), with time spent in Chores providing the most noticeable example, where across the BODE index, reductions totaling 206 min/d were observed. These reductions were principally offset by the increases in Screen (156 min/d) and/or Quiet time (131 min/d).

Despite these differences, recommendations surrounding activities typical of COPD populations (e.g., Chores and sedentary behaviours) are conspicuously absent in international COPD-specific guidelines [39]. In Lewthwaite et al.’s recent review [39] of 35 COPD specific clinical practice guidelines, no COPD-specific clinical practice guidelines included recommendations for either sleep or sedentary behaviours [2,40]. The broader concept of physical activity was addressed in 21 of the included guidelines, but guidance generally mirrored general adult recommendations (e.g., 30 min/d or 150 min/wk of MVPA). Consistent with previous reports, our compositional models predicted little MVPA engagement (24 min/d of MVPA at BODE 0) and small changes in MVPA, totaling only 16 min/d across BODE index scores, indicating that the current recommendations may be unachievable to most with COPD. 

Increasingly researchers are looking towards a more personalised approach to COPD management (e.g., tailored rehabilitation programs and/or individualised self-management), so understanding how people with COPD use their time not only at a population level, but also at the level of the individual, is important to identify outcomes that are both population-appropriate and meaningful. This study identifies use-of-time data as a potential outcome measure that can be used when developing personalised interventions and/or in further research that is aimed at exploring the efficacy of interventions developed for people with COPD. Although cross-sectional, our models imply that there is an association between functional capacity and COPD severity. Our models also suggest increases in screen-based and other quiet activities are likely to occur at the expense of physically demanding activities, such as chores. This presents clinicians with an opportunity, enabling them to support people with COPD to make choices regarding the types of activities that they choose to fill this void. Promotion of interventions that specifically target these aspects of time use, for example programs that promote social interaction, chores or walking may prove beneficial. 

To our knowledge, this is the first study in the COPD literature to treat activity and/or use-of-time data using a compositional approach. Traditionally, analyses have omitted one or more compositional components from their analyses. Selective omission of individual components presents statistical challenges, as inherent co-dependency exists between individual components of a given composition (i.e., increase in one component [e.g., Screen time] leads to decreases in one or more of the remaining compositional components [e.g., Chores, Sleep, or Sports/exercise]) [35,41]. CoDA acknowledges the closed nature of activity data, the resultant constraints, and takes into account the malleable nature of the activity patterns in the ensuing analyses.

The current study is not without limitations. Our cross-sectional design had a modest sample size and a number of participants recorded minimal or no time in some the compositional model components (e.g., four individuals recorded no daily MVPA on accelerometry and nine individuals reported no daily Sports/exercise on MARCA-recall). Despite demonstrating strong reliability and validity against accelerometry in adults and people with COPD, MARCA data may be influenced by poor recall or social desirability bias. We mitigated as many of these conditions as possible, recruiting from a single metropolitan area, over multiple seasons, but factors, such as familial living arrangements, may be residual confounders. Additionally, MARCA-derived energy expenditure estimates are based on healthy adult data [42] and may misrepresent the actual energy requirements of this population. Finally few participants had high BODE index scores (median BODE = 3, range 0–9, with *n* = 10 scores ≥ 7), and data extrapolation to BODE index score 10 is tentative.

## 5. Conclusions

People with COPD spent the majority of their day engaging in activities that required a low expenditure of energy (Screen time, sitting quietly). Little, if any, time was spent in formal Sports/exercise. Our modelled data suggests the activity patterns of people with COPD are likely to be closely associated with the severity of their disease, as reflected by the BODE index score. Compositional regression models predicted that people with more severe disease were predicted to have greater Screen and Quiet time occurring at the expense of chores. This study’s findings suggest that certain activities act as key indicators of disease severity (e.g., Chores and walking). Time–use patterns may provide a basis for planning interventions relative to the severity of COPD

## Figures and Tables

**Table 1:** Baseline data—Physiological.

	Characteristic	Females (*n* = 57)	Males (*n* = 84)	Total Sample (*n* = 141)
Demographics	Age	69.2 (8.1)	71.6 (8.1)	70.6 (8.1)
Spirometry	% female (n)	-	-	40% (57)
	BMI (kg m^−2^)	28.5 (7.3)	27.7 (5.5)	28.0 (6.2)
	FEV_1_ (%pred)	50.4 (15.4)	49.6 (17.5)	49.9 (16.6)
	FVC (%pred)	81.8 (18.2)	83.2 (19.5)	82.7 (19.0)
	FEV_1_/FVC (%)	46.6 (13.1)	42.2 (14.7)	43.9 (14.2)
6 min walk test	6MWD (m)	356 (132)	380 (136)	370 (134)
Exertional breathlessness	mMRC Grade 0 (n) Grade 1 (n) Grade 2 (n) Grade 3 (n) Grade 4 (n)	1.8 (1.1) 1 30 9 12 5	2.0 (1.3) 9 26 16 18 15	2.0 (1.2) 10 56 25 30 20
BODE index score		3.0 (1.8)	3.2 (2.3)	3.1 (2.1)
COTE index score		2.0 (2.1)	1.5 (1.9)	1.7 (2.0)

All values are expressed as Mean (SD) unless otherwise stated. n—number, BMI—Body Mass Index, FEV_1_—forced expiratory volume in 1 s, FVC—forced vital capacity, FEV_1_/FVC ratio—Forced expiratory volume in 1 s (FEV_1_) expressed as a percentage of Forced vital capacity (FVC), 6MWD—six minute walk distance, mMRC—modified Medical Research Council dyspnoea questionnaire, BODE index—the Body mass, airflow Obstruction, Dyspnoea and Exercise index, COTE index—COPD-specific COmorbidity TEst Index.

**Table 2:** Baseline MARCA recall use of time data presented using arithmetic means for super- and macrodomains (in min/d).

Super-Domain	Macro-Domain	Females (*n* = 53)	Males (*n* = 82)	Total (*n* = 135)
Chores		200 (100)	168 (94)	181 (97)
	Inside chores	183 (89)	141 (79)	158 (85)
	Outside chores	16 (28)	28 (51)	23 (44)
Household administration		67 (72)	65 (48)	66 (58)
	Employment	25 (62)	17 (30)	20 (45)
	Study	7 (23)	3 (11)	4 (17)
	Passive transport	35 (26)	45 (36)	41 (32)
Quiet time		157 (92)	170 (92)	165 (92)
	Reading	69 (63)	76 (76)	73 (71)
	Non-reading	88 (72)	94 (65)	92 (67)
Screen time		266 (105)	295 (130)	284 (121)
	Television viewing	236 (109)	243 (110)	240 (109)
	Computer Use	29 (47)	50 (72)	42 (64)
Self-care		139 (27)	139 (27)	139 (27)
	Eating	85 (21)	90 (20)	88 (20)
	Grooming/ablutions	54 (21)	49 (14)	51 (17)
Sleep		483 (78)	480 (76)	481 (77)
Socio-cultural		106 (70)	97 (56)	100 (62)
	Communication	75 (45)	81 (52)	79 (49)
	Cultural	17 (51)	6 (19)	11 (36)
	Socialising	14 (27)	9 (18)	11 (22)
Sports/exercise		22 (28)	26 (31)	25 (30)
	Play	1 (7)	2 (9)	2 (8)
	Sport	5 (17)	7 (19)	6 (18)
	Active transport	16 (19)	17 (19)	17 (19)

All values are expressed as mean (SD) and represent time spent in each category in minutes per day (min/d). *n* = number.

**Table 3:** The relationship between the BODE index and relative time in energy-expenditure band (Model 1 & 2) and time-use components (Model 3).

		Estimate (95% CI)	*t* Value	*p*	Adj.R^2^
	Pivot Coordinate (Relative to Remaining)				
Model 1	Sleep	0.11 (0.07; 0.14)	5.9	<0.0001 *	0.26
	Sedentary	0.15 (0.11; 0.19)	7.1	<0.0001 *	0.31
	LPA	−0.02 (−0.05; 0.02)	−1.1	0.282	−0.02
	MVPA	−0.24 (−0.32; −0.15)	−5.4	<0.0001 *	0.21
Model 2	Sleep	0.07 (0.05; 0.09)	6.3	<0.0001 *	0.25
	Sedentary	0.13 (0.10; 0.15)	8.8	<0.0001 *	0.38
	LPA	−0.02 (−0.04; 0.02)	−0.9	0.344	−0.02
	MVPA	−0.18 (−0.22; −0.14)	−8.3	<0.0001 *	0.34
Model 3	Chores	−0.15 (−0.21; −0.09)	−5.1	<0.0001 *	0.18
	Sleep	−0.09 (0.00; 0.05)	−2.5	0.014 *	0.07
	Sports/exercise	0.11 (−0.17; −0.01)	3.8	<0.0001 *	0.02
	Quiet time	0.09 (0.05; 0.17)	3.9	<0.0001 *	0.12
	Screen time	0.04 (0.04; 0.14)	3.5	0.001 *	0.06
	Self-care	0.03 (0.02; 0.06)	2.3	0.022 *	0.08
	Household administration	0.07 (−0.16; −0.02)	2.5	0.015 *	0.03
	Sociocultural	−0.09 (0.01; 0.12)	−2.3	0.025 *	0.03

Adj.R^2^—adjusted r squared value, LPA—Light physical activity, MVPA—moderate to vigorous physical activity. *—indicates statistically significant results. The model includes terms for COTE index score, socio-economic status (IRSD), smoking status, age, and sex. The estimate corresponds to the first isometric log-ratio coordinate only. It therefore provides a numerical value reflecting the slope of a given component relative to all remaining components collectively (e.g., sleep relative to the remaining day). This value represents a proportional change for each one-point change in BODE index score. It should be considered in conjunction with the observed *p* value.
